# The usefulness of electrocardiogram in the recognition of cardiac transplant rejection

**DOI:** 10.1002/clc.23825

**Published:** 2022-04-08

**Authors:** Huzaifa A. Cheema, Abia Shahid, Muhammad Ayyan, Muhammad Ehsan

**Affiliations:** ^1^ Department of Medicine King Edward Medical University Lahore Pakistan

To the Editor,

We read with great interest but some consternation the recent meta‐analysis by Hashim et al.^1^ in which they evaluated the role of electrocardiogram (ECG) as a noninvasive measure of early allograft rejection in cardiac transplant recipients. We find several methodological flaws in this review that warrant discussion. First, the authors aimed to carry out a systematic review of diagnostic test accuracy but they did not use any diagnostic accuracy metrics that are commonly used or recommended by the *Cochrane Handbook for Systematic Reviews of Diagnostic Test Accuracy*.^2^ The most commonly used metrics are sensitivity and specificity but others such as areas under receiver operating characteristic curves and diagnostic odds ratios can also be used.^3^ Instead, the authors compared ECG changes in patients with different severity of transplant rejection. This is akin to using an inches ruler to measure meters. The results of this meta‐analysis, hence, provide little insight into the performance of ECG as a diagnostic test for transplant rejection compared to a reference standard. The authors should pool sensitivity and specificity using recommended methods.

Second, the authors provide vague eligibility criteria followed by the inclusion of human studies as well as animal studies. Animal studies in a systematic review of human studies serve no purpose and only function to further weaken the results of this study.^4^ The authors also fail to acknowledge and delineate between the different species in their studies; referring to animals as “patients” is equivocal.

Finally, the authors framed their conclusion as rather wishful words that are misleading. Their results returned no significant association between ECG changes and transplant rejection but they termed it as a “good choice for screening” and “potential to reduce the number and severity of rejection episodes.” To avoid this discordance, the authors should align their conclusions with their results.

## CONFLICTS OF INTEREST

The authors declare no conflicts of interest.
